# Scheffersomyces tanahashii sp. nov., isolated from the cocoon wall of the stag beetle Prosopocoilus astacoides blanchardi

**DOI:** 10.1099/ijsem.0.006720

**Published:** 2025-03-13

**Authors:** Matan Shelomi

**Affiliations:** 1 Department of Entomology, National Taiwan University, Taipei, Taiwan, ROC

**Keywords:** Debaryomycetaceae, *Lucanidae*, one new taxon, *Saccharomyceta*, yeast, Taiwan

## Abstract

A previous investigation of symbiotic yeasts associated with the stag beetle *Prosopocoilus astacoides blanchardi* isolated strains of the genus *Scheffersomyces* from the cocoon walls, larval midgut, larval hindgut and larval tunnels. Phylogenetic analysis of the D1/D2 domains of the LSU rRNA gene sequences revealed identical sequences, indicating that they belonged to the same species, but suggested that the species was new. In this study, sequence analysis and physiological characterization identified a representative strain of these beetle-associated yeasts as a novel species in the genus *Scheffersomyces*. The sequence similarities of the concatenated LSU domains and internal transcribed spacer regions indicated that strain BCRC 23563^T^ forms a well-supported and distinct species in the xylose-fermenting *Scheffersomyces* subclade, with the sequences for each gene differing in nt substitutions from those of previously described related species by at least 1.06% and 2.7% respectively. The physiological characteristics of the novel species were also distinct from those of the closely related described species, though it could still process xylose as is expected of stag beetle-associated *Scheffersomyces* symbionts. Based on the data, a novel yeast species, *Scheffersomyces tanahashii* sp. nov., is proposed to accommodate this strain. The holotype is BCRC 23563^T^ (ex-type strains NBRC 116731 and NCYC 4470). The MycoBank accession number is 857608.

## Data Summary

Sequence data generated by this research is available at GenBank, accession numbers PQ349791–PQ349795 and PQ349796–PQ349800. The authors confirm all supporting data, code and protocols have been provided within the article or through supplementary data files.

## Introduction

The genus *Scheffersomyces* was proposed by Kurtzman and Suzuki [1] along with *Babjeviella*, *Meyerozyma*, *Millerozyma* and *Priceomyces* as a new clade in a new family, *Debaryomycetaceae*, formed from the genera *Debaryomyces*, *Lodderomyces*, *Spathaspora*, *Yamadazyma* and selected species of *Pichia* and *Candida* solely on the basis of concatenated D1/D2 large and small subunit rRNA gene sequences. Species in this family all form coenzyme Q-9. *Scheffersomyces* species generally produce one to two hat-shaped ascospores released soon after formation. They typically have spherical to elongate budded cells and form pseudohyphae but not true hyphae [1,2]. The genus ferments glucose and some other sugars, in particular d-xylose: *Scheffersomyces* holds the majority of the d-xylose-fermenting yeasts of interest to the biofuel [3] and bioethanol [4,5] industries. A noted exception unable to ferment d-xylose is *Scheffersomyces spartinae*, first isolated from marshland [6]. Since its description, novel xylose-fermenting species have been added on the basis of phylogenetic analysis [7]. With the exception of *Sc. spartinae*, which is only known from brackish water [2,6, 8, 9], the other valid species of *Scheffersomyces* were all discovered either in rotting wood [10,16] or in insects that eat rotting wood such as wood cockroaches (*Blattodea*: *Cryptocercidae)* [17], bess beetles (*Coleoptera: Passalidae*) [18], darkling beetles (*Coleoptera*: *Tenebrionidae*) [18], longhorn beetles (*Coleoptera: Cerambycidae*) [12] and stag beetles (*Coleoptera: Lucanidae*) [18,20]. In the original paper describing the genus [1], two varieties of the species *Candida shehatae* were reclassified into two species, *Candida insectosa* and *Candida lignosa*, based with one isolated from a Cerambycidae beetle and another from the wood it infested [21,22], and a later paper would separately classify these and the original species as three separate species: *Scheffersomyces shehatae*, *Scheffersomyces insectosa* and *Scheffersomyces lignosus* [7]. The placement of *Sc. spartinae* in the genus in the Kurtzman and Suzuki paper, where it was the weakly supported basal species of the genus [1], remains controversial [8]. There are currently 22 valid species of *Scheffersomyces*, plus two with unclear status due to nomenclatural issues arising from non-adherence to the Melbourne Code [12], *Scheffersomyces gosingicus* isolated from forest soil [23] and *Scheffersomyces lignicola* isolated from insect frass [24].

Stag beetles have wood-feeding larvae highly dependent on fungi to digest their food [25]. These fungi are typically xylose-fermenting yeasts [26], most commonly from the genus *Scheffersomyces* [27,28], some of which remain undescribed [27]. These symbiotic yeasts can be vertically transmitted by females that store them in their mycangia and deposit them when ovipositing [29]. As the larvae develop, they form a cocoon wall from their faeces incorporating the symbiotic yeasts, and when females emerge, they evert their mycangium and rub it on the cocoon wall to acquire the yeasts and continue the transmission cycle [19,26, 30]. In a 2023 paper published in the *Coleopterists' Bulletin*, several yeasts were isolated from the mycangia, larval gut and larval tunnels of wild and lab-reared *Prosopocoilus astacoides blanchardi* (*Coleoptera: Lucanidae*) from Taiwan [31]. The isolated yeasts varied greatly among individuals, suggesting that the associations between yeast symbionts and these beetle hosts do not follow a one-to-one relationship with essential vertical transmission as had been documented for other stag beetle species [28]. Among the microbes cultured were strains of *Scheffersomyces* and *Spathaspora*. The *Scheffersomyces* isolates, regardless of source, did not show significant divergence amongst themselves based on 28S rRNA sequencing and were paraphyletic to a monophyletic clade containing the type species of the genus, *Scheffersomyces stipitis* [1]; *Scheffersomyces illinoinensis*, isolated from rotting hardwood [7]; *Scheffersomyces segobiensis*, isolated from soil [1,32]; *Scheffersomyces stambukii*, isolated from rotting wood [11]; and *Scheffersomyces titanus*, isolated from another stag beetle, *Serrognathus titanus* [20]. All the isolated *Scheffersomyces* yeasts – over 20 isolates from larval midguts (strains L10MG, L11MG, L14MG, L15MG, L16MG and L19MG), larval hindguts (L2HG-2, L11HG, L20HG-1, L20HG-2 and L21HG), larval tunnel walls (LT3, LT8, LT9, LT10, LT11, LT14, LT16 and LT17), cocoon walls (CW2, CW8, CW9, CW12, CW15 and CW20) and one adult mycangium (A14MYC) – were identified on the basis of D1/D2 sequence similarity and phylogenetic analysis as strains of the same species, and likely one that was novel to science [31]. Further efforts to differentiate the species and confirm these identities have only now been completed. Phylogenetic analysis based on the concatenated sequences of the internal transcribed spacer (ITS) regions and D1/D2 domains of the LSU rRNA gene and physiological characterization indicates clearly that the *Scheffersomyces* species isolated from the beetles, exemplified by strain CW2, is a novel species, hereby proposed as a novel species, *Scheffersomyces tanahashii* sp. nov.

## Methods

### Yeast isolation and culture

Strains were isolated and purified from *P. astacoides blanchardi* and their habitats reared at the Department of Entomology at National Taiwan University (25.019537 N 121.543440 E) from larvae collected at Tai'an Township, Taiwan, as described in the original paper [31]. Strain CW2, a representative isolate among over 20 of the putative new *Scheffersomyces* species, was isolated from wood pieces taken from the cocoon wall of a *P. astacoides blanchardi* adult after it had eclosed, with sampling from the end closest to the abdomen tip (the area where the adult female would rub her everted mycangium on to re-acquire symbiotic yeasts). The wood sample was placed in a 1.5 ml Eppendorf tube with 160 µl of sterilized PBS (see the original paper for the recipe), homogenized, diluted and loaded onto 90 mm Petri dishes of potato dextrose agar (HiMedia^®^, India) and 100 mg ml^-1^ chloramphenicol (Bioman Scientific Co., Ltd., Taipei, Taiwan) incubated aerobically at 27 °C in the dark for 2–3 days. Colonies were purified on potato dextrose agar. All isolated yeast strains were preserved in 50% glycerol stocks and stored at −80 °C, and a few isolated and purified yeasts were sent to the Bioresource Collection and Research Center (BCRC) of the Food Industry Research and Development Institute in Hsinchu, Taiwan, for permanent strain preservation in a metabolically inactive state and identification testing. Unfortunately, due to a catastrophic failure of the -80 °C freezer in which the glycerol stocks were stored, no other representatives of the putative new *Scheffersomyces* species could be recovered other than CW2, now BCRC 23563^T^. Whilst single isolate descriptions are not favoured, they are allowed [33,34]. Subsequent analyses were performed on ex-type cultures revived from BCRC 23563^T^, and isolates thereof were also publicly deposited in the NITE Biological Resource Center (NBRC) in Chiba, Japan, and the National Collection of Yeast Cultures (NCYC) in the UK.

### Morphological, physiological and biochemical characteristics

To delineate species as per the phenotypic species concept [35], strain BCRC 23563^T^ was compared along with other strains isolated from other *Prosopocoilus* species in the BCRC collection and compared to known data for related *Scheffersomyces* species. To identify strains on the basis of morphological characteristics, strains were cultured in yeast malt (YM) broth (yeast extract, 3.0 g; malt extract, 3.0 g; peptone, 5.0 g; dextrose, 10.0 g; distilled water, 1.0 l), then YM agar (YM broth recipe plus 20.0 g agar) at 25 °C for 3 days. To test for pseudohyphae, strains were cultured on Dalmau plates at 26 °C for 7 days. To test for sporulation, in addition to YM and Dalmau, strains were cultured on diluted V8 agar (1:19) at 20 °C for up to 14 days. Biochemical profile analysis with a VITEK 2 COMPACT (bioMérieux, France) was done according to the manufacturer’s instruction using the VITEK 2 YST ID card and VITEK 2 COMPACT identification software v9.02. The biochemical tests examined carbon source utilization, nitrogen source utilization and enzyme activities involving the following compounds: 2-keto-d-gluconate, acetate, alpha-glucosidase, amygdalin, arbutin, arginine, beta-*N*-acetylglucosaminidase, d-cellobiose, d-galactose, d-galacturonate, d-gluconate, d-glucose, d-maltose, d-mannose, d-melezitose, d-melibiose, d-raffinose, d-sorbitol, d-trehalose, d-turanose, d-xylose, dl-lactate, erythritol, aesculin, gamma-glutamyl transferase, glucuronate, glycerol, l-arabinose, l-glutamate, l-lysine arylamidase, l-malate, l-proline, l-rhamnose, l-sorbose, lactose, leucine arylamidase, methyl-A-d-glucopyranoside, *N*-acetyl-glucosamine, nitrate, PNP-*N*-acetyl-BD-galactosaminidase, R-rhamnose, saccharose/sucrose, sodium citrate, tyrosine arylamidase, urease and xylitol. The results of those tests are summarized in Table 1.

**Table 1.** Selected phenotypic test results for characteristics distinguishing different *Scheffersomyces* strains. CW2: *Sc. tanahashii* sp. nov. BCRC 23563^T^. (a) *Scheffersomyces cryptocercus* NRRL Y-48824^T^ [17]. (b) *Scheffersomyces henanensis* CICC 1974^T^ [10]. (c) *Sc. illinoinensis* NRRL Y-48827^T^ [7]. (d) *Sc. lignosus* CBS 4705^T^ [22]. (e) *Sc. segobiensis* CBS 6857^T^ [44]. (f) *Sc. shehatae* CBS 5813^T^ [22]. (g) *Sc. stipitis* BCRC 21775^T^. (h) *Sc. stipitis* CBS 5773^T^ [44]. +: positive; −: negative; v: variable; w: weakly positive; d: delayed positive; [blank]: not tested

	CW2	A	B	C	D	E	F	G	H
Pseudohyphae	+	+	+	+	+	w	+	+	+
Ascospores	−	−	+	−	−	+	−	+	+
Nitrate reduction	−	−	−	+	−	−	−	−	−
Assimilation test									
Citrate (sodium)	+	−	+	+	+	+	+	+	+
d-Cellobiose	+	+	+	+	+	+	+		+
d-Galactose	−	+	+	+	+	+	+	+	+
d-Glucose	+	+	+	+	+	+	+	+	+
d-Maltose	+	+	+	+	+	+	+	+	+
d-Melezitose	−	+	d	+	+	−	+		+
d-Melibiose	−	+	−	+	−	−	−	−	−
d-Raffinose	−	−	−	−	v	−	v	−	−
d-Trehalose	+	+	+	+	+	+	+	+	+
d-Xylose	+	+	+	+	+	+	+	+	+
dl-Lactate	−	−	−	+	+	−	−		+
Erythritol	−	−	d	+	v	−	v		+
Glucuronate	v	−			−		v	−	
Glycerol	−	−	+	+	+	+	+		+
l-Arabinose	−	w	−	+	v	−	v	−	v
l-Rhamnose	+	−	+	+	−	−	−	+	+
l-Sorbose	−	+	−	−	+	+	v	−	v
Lactose	−	−	−	−	v	−	v	−	v
*N*-Acetyl-glucosamine	−	+	−	+	+	+	+	+	+
Saccharose/sucrose	+	+	+	+	v	+	+	v	+
Xylitol	−	−			v		v	−	

### DNA sequencing and phylogenetic analysis

DNA sequencing and phylogenetic analyses were performed to determine genetic relationships within the yeast species as per the phylogenetic species concept [35], which was the concept applied to justify creating the genus *Scheffersomyces* in the first place [1] and remains the most important species concept used when adding new species to the genus [7,10, 12, 20]. For phylogenetic analysis, Sanger sequencing of the D1/D2 region of the LSU rRDNA and the complete ITS region was done on clones isolated on the YM agar at 26 °C for 2–3 days. Genomic DNA was extracted using a FavorPrep^™^ Blood/Cultured Cell Genomic DNA Extraction Kit (Taiwan) as per the manufacturer’s instructions. The primers used for LSU sequencing were the standard primers NL1/F63 (5′-GCA TAT CAA TAA GCG GAG GAA AAG-3′) and NL3/LR3 (5′-GGT CCG TGT TTC AAG ACG G-3′) [36], and for ITS, the standard primers ITS1 (5′-TCC GTA GGT GAA CCT GCG G-3′) and ITS4 (5′-TCC TCC GCT TAT TGA TAT GC-3′) were used [36,37]. The PCR polymerase was Biomate^™^ Taq DNA polymerase (Taiwan). The nt sequence of each PCR product was determined by Sanger sequencing performed at Tri-I Biotech Inc. (Taiwan) on an ABI 3730xl DNA Analyzer. The resulting LSU and ITS barcode sequences for BCRC 23563^T^ have been uploaded to GenBank with accession numbers PQ349791 and PQ349796, respectively. Up to 100 complete LSU (D1/D2) and ITS sequences from type strains of *Scheffersomyces* and related genera identified from a blast search of the GenBank database were downloaded [38]. Sequences were aligned with Clustal Omega multiple sequence alignment with an online web server [39]. Using mega x [40], the ends and gappy regions were trimmed. Next, the aligned LSU and ITS sequences were concatenated. A maximum-likelihood tree was made from the alignment using the IQTREE [41,42] v2.3.6 web server [http://iqtree.cibiv.univie.ac.at/] with 1000 bootstrap alignments to assess the confidence levels of the clades, with *Saccharomyces cerevisiae* used as an outgroup. Trees were visualized with FigTree v1.4.4 [http://tree.bio.ed.ac.uk/software/figtree/].

## Results and discussion

BCRC 23563^T^ was isolated from *P. astacoides blanchardi* [31] along with other *Scheffersomyces* and related yeasts from the same beetle and the closely related *Prosopocoilus motschulskii*. Using only LSU and ITS sequencing corroborated by phenotype as described above, several of these strains could be conclusively identified to species. The mycangium of wild-caught female *P. motschulskii* isolated a strain of *Sc. segobiensis* (BCRC 23557 and NBRC 116728) and a strain of *Sc. stipitis* (BCRC 23558 and NBRC 116729) identified with 100% sequence similarity for both 26S and ITS genes. The mycangium of a lab-reared, newly eclosed female *P. astacoides blanchardi* isolated a strain of *Sc. stipitis* (BCRC 23559 and NBRC 116730) also with 100% sequence similarities. From a *P. astacoides blanchardi* larval midgut, a strain was isolated and identified as *Spathaspora elongata* (BCRC 23564, NBRC 116732 and NCYC 4471) partly on the basis of the LSU sequence having 99.65% sequence similarity to that of *S. elongata* NYNU 18115^T^, and the ITS sequences having 100% sequence similarity. I give these examples to demonstrate the effectiveness of the above methods and the chosen phylogenetic species concept to identify *Prosopocoilus*-associated yeasts to species, if they are of a previously described species, and, by the same logic, justify the observation that BCRC 23563^T^ is novel. These same methods have also been sufficient to differentiate and describe species in *Debaryomycetaceae* as recently as the past year [43].

The blastn search of the type specimens in the GenBank rRNA/ITS databases found differences in the BCRC 23563^T^ barcode sequences compared to those of published types. The LSU rRNA sequence showed a 99.12% sequence similarity to that of *Sc. shehatae* NRRL Y-12858^T^ (five nt substitutions and one gap) and 99.12% sequence similarity to that of *Sc. lignosus* ATCC 58779^T^ (five nt substitutions and two gaps). If performing a blastn search of the type specimens in the GenBank standard core nt database, strain *Sc. cryptocercus* R6P1^T^ also appears with a 99.12% sequence similarity (five nt substitutions and no gap). Whilst there are some exceptions, according to the genetic species concept, for this region of the LSU, ‘conspecifics may differ by up to three nucleotide substitutions and species differ with six or more substitutions’ [35,36, 44], which puts the LSU sequence for BCRC 23563^T^ just barely in the boundary area with room for interpretation. Prior authors [22] have stated that *Sc. shehatae* cannot be differentiated from *Sc*. *insectosa* or *Scheffersomyces lignosa* on the basis of LSU and that ITS sequencing at a minimum must be added to differentiate these species. The ITS sequence of BCRC 23563^T^ showed 97.30% sequence similarity to those of both *Sc. illinoinensis* NRRL Y-48827^T^ (12 nt substitutions and 4 gaps) and *Sc. stipitis* ATCC 58376^T^ (13 nt substitutions and 4 gaps). A commonly used percent similarity cutoff for ITS sequencing for fungal species identification is ~98.5% [45,47]. Some authors have used an ITS sequence similarity cutoff of 98.41% between different yeast species and 96.31% for different yeast genera and an LSU sequence similarity cutoff of 99.51% and 97.11% for different yeast species and genera, respectively [48]. Given that the two different barcodes did not point to the same species as closest relatives and that the ITS sequence similarity is beyond any used cutoff for a new species, I am inclined to interpret the >3 nt substitutions in the LSU sequence as suggesting BCRC 23563^T^ is not any other known *Scheffersomyces* species as per the genetic species concept [35].

**Fig. 1.**
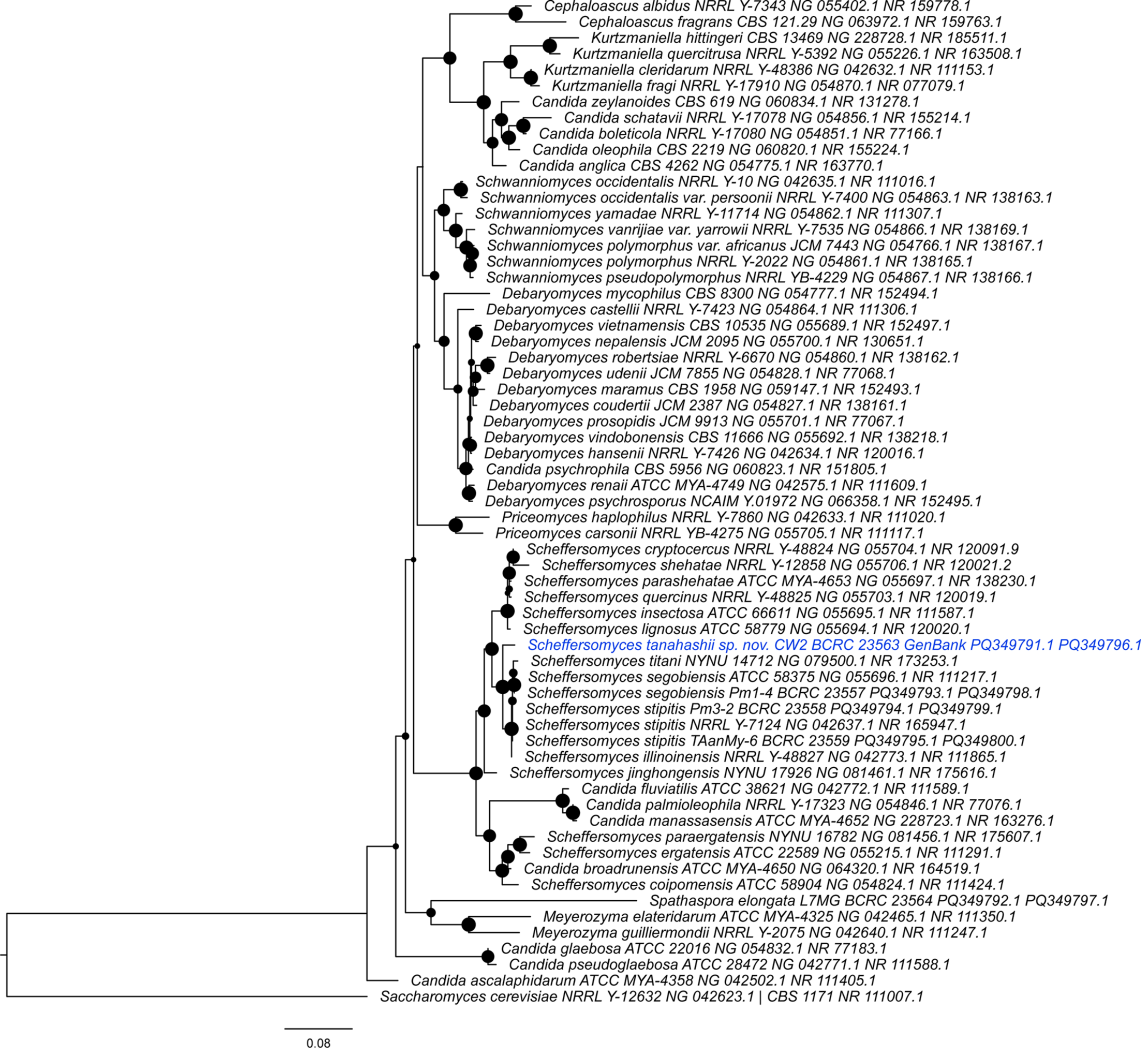
The maximum-likelihood tree for the concatenated LSU and ITS sequences made with IQTREE v1.6.12 [41,42] with ultrafast bootstrapping [52] and the best-fit model GTR+F+I+G4 as chosen by ModelFinder [53]. The following species and strains are written with the GenBank accession numbers for the LSU and ITS sequences, respectively. Node circles correspond to bootstrap values, with larger circles indicating higher support from 0% to 100%. Figure prepared with FigTree v1.4.4.

Phylogenetic analysis of concatenated LSU and ITS sequences of the *Scheffersomyces* and related yeasts, with *Saccharomyces* as an outgroup, supported the novel species designation for BCRC 23563^T^. The use of concatenated gene trees has precedence in recent *Scheffersomyces* species descriptions [11,12] and is reported to be more accurate than consensus trees of separate gene phylogenies [49,50]. The maximum-likelihood tree of the concatenated genes (Fig. 1) placed BCRC 23563^T^ as a distinctly separate taxon to a clade containing *Scheffersomyces stitipis*, *Sc. illinoinensis*, *Sc. segobiensis* and *Scheffersomyces titani*. This can be compared to the other, aforementioned *Scheffersomyces* strains isolated from *Prosopocoilus* spp. with 100% sequence similarities to known species, which formed close sister groups with their conspecifics. The phylogenetic data places the new species BCRC 23563^T^ within what Urbina and Blackwell describe as the xylose-fermenting subclade of *Scheffersomyces* [7], producing a phenotype prediction that ended up confirmed.

**Fig. 2.**
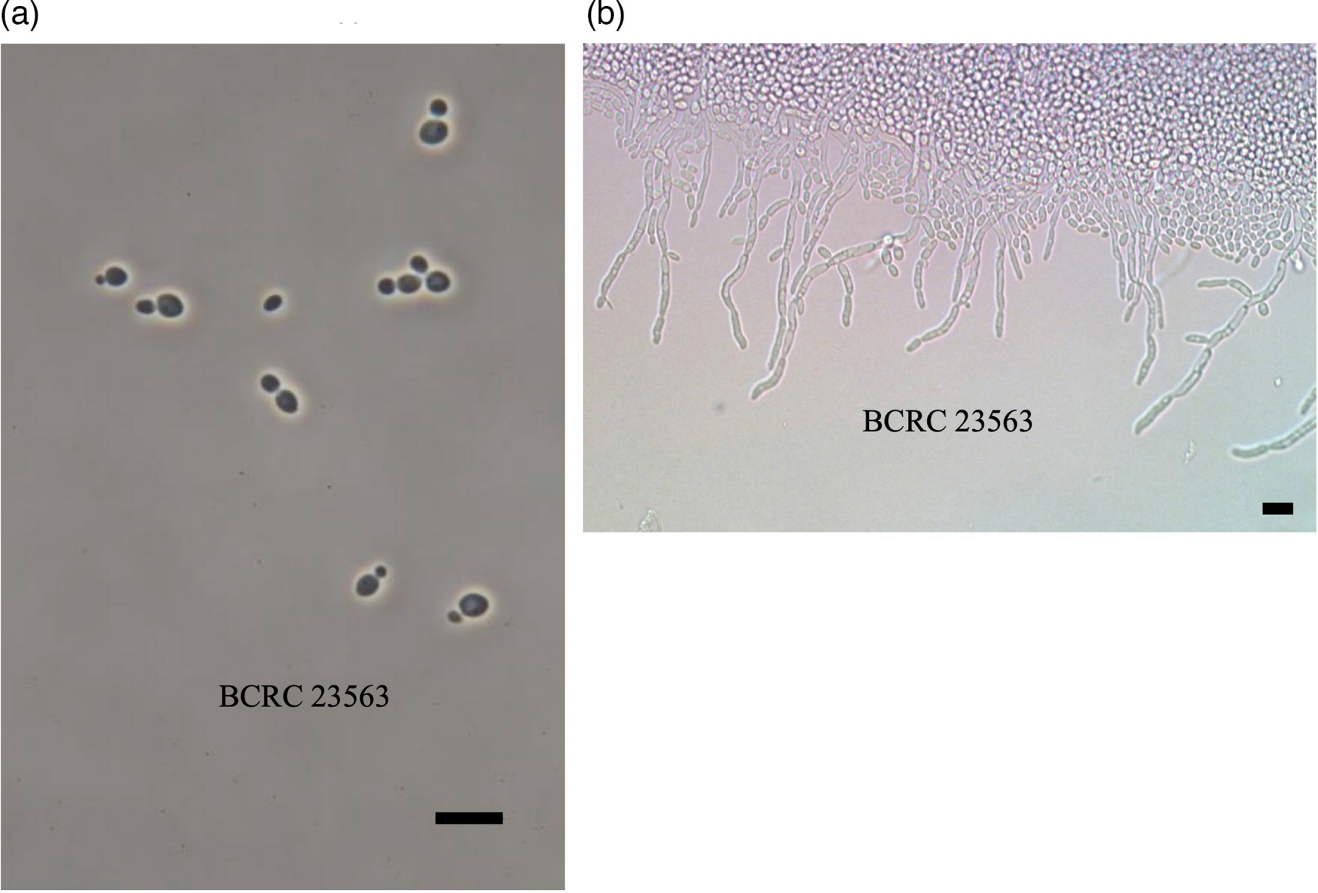
Micrographs of strain 23563^T^. All scale bars are 10 µm. (a) Yeasts after growth in YM broth. (c) Positive pseudohyphae test after culturing on Dalmau plates at 26 °C. Image (a) was made with a phase contrast microscope (Eclipse E600, Nikon) and Canon Digital EOS Rebel T5i Camera DS126431, using EOS Utility 2 v2.14.20a to take the images and AxioVision LE v4.8.2.0 to generate the scale bar. Image (b) used a differential interference contrast microscope (MICROPHOT-FXA, Nikon) with a Nikon FDX-35 Camera and the MShot Digital Imaging Analysis System v1.1.6 to take the pictures and add a scale bar.

Regarding phenotype, strain BCRC 23563^T^ (ex-type NBRC 116731 and NCYC 4470) has spherical to ovoid cells with a size of 2–4.5×2–7 µm after cultivating in YM broth for 3 days (Fig. 2a). They proliferate asexually by multipolar budding and formed single or paired cells. In broth, it is non-flocculent with no ring or pellicle. On solid YM agar media, the colonies are off-white with a smooth, dull surface. No ascospores are evident after growth on any media. The pseudohyphae test is positive after 1 day on the Dalmau plate (Fig. 2b), but no true hyphae are formed. The carbon assimilation tests summarized in Table 1 show some distinctive attributes separating BCRC 23563^T^ from its closest phylogenetic relatives. These physiological and biochemical test results show the overall closest similarity to the published characteristics of *Sc. shehatae* CBS 5813^T^ [22,44] and *Sc. henanensis* CICC 1974^T^ [10]. However, BCRC 23563^T^ differs from the former in its ability to degrade l-rhamnose [22] and from the latter in its lack of ascospores after growth on any of the media (Table 1). It differs from all listed *Scheffersomyces* species in an inability to assimilate d-galactose. BCRC 23563^T^ could thus be differentiated from related species [35]. When combined with the genetic and phylogenetic data, the results surpass the minimally accepted evidence for a new species [33]. In accordance with the Shenzhen Code [51], it is reasonable to place this species in the genus *Scheffersomyces*.

In this study, I identify a strain hypothesized to be a novel species in a previous paper describing its isolation from the cocoon wall of a *P. astacoides blanchardi* stag beetle and preliminary identification work with LSU sequence analysis [31]. That study identified the strain to the genus *Scheffersomyces*, for which LSU sequences alone are not sufficient to differentiate between species [7]. Here, the strain is identified as a new species based on phenotypic differences and, in particular, genetic differences in the LSU and ITS gene sequences passing accepted thresholds separating new species [48]. As many species of *Scheffersomyces* have been isolated from wood-feeding insects or habitats associated with them, the finding of one in *P. astacoides blanchardi* was not surprising. Based on the original published data as well as previous findings in beetle-associated *Scheffersomyces* [28], BCRC 23563^T^ represents a species, which the name *Scheffersomyces tanahashii* sp. nov. is proposed to accommodate, that can be found in the *P. astacoides blanchardi* larval midgut, hindgut, tunnel and cocoon wall, even if the holotype was isolated from the latter. Whilst *Scheffersomyces* species dominated in that study, some of the sampled beetle larvae guts were predominantly colonized by *Spathaspora* instead, and 15 other genera of fungi were also reported [31]. That multiple species of yeast can be associated with the same species of stag beetle in the same location in Taiwan, suggesting that *Lucanidae* species do not necessarily form specific symbiotic relationships with a single species or genus of yeast [28]. Instead, a number of yeasts associated with rotting wood, such as several different *Scheffersomyces* species, could potentially colonize the gut of larvae and assist with digestion and/or colonize the mycangium of recently emerged adults and potentially be passed vertically to the next generation [26,30]. It remains unclear what difference in benefits, if any, *Sc. tanahashii* would provide relative to other species of *Scheffersomyces*, or a species of another genus for that matter. What the discovery of *Sc. tanahashii* does suggest is that more species of wood- and/or insect-associated *Debaryomycetaceae* remain to be described.

## Description of *Scheffersomyces tanahashii* Shelomi sp. nov.

*Scheffersomyces tanahashii* (ta.na.ha’shi.i N.L. gen. n. *tanahashii*, referring to Masahiko Tanahashi, in recognition of his research on stag beetle-associated yeasts).

After 3 days of growth on YM broth at 25 °C, cells are spherical to ovoid (2–4.5×2–7 µm) and occur singly or in pairs (Fig. 2). They proliferate asexually by multipolar budding and form single or paired cells. On YM agar after 3 days at 25 °C, the streak culture is off-white, raised, with a smooth surface and has an entire margin. Pseudohyphae are observed on Dalmau plates after 1 day at 25 °C. True hyphae, ascospores and asci are not evident when the strains are incubated on YM, Dalmau or V8 media. Carbon assimilation is positive for d-cellobiose, d-glucose, d-maltose, d-trehalose, d-xylose, l-rhamnose, sucrose and sodium citrate; negative for d-galactose, d-melezitose, d-melibiose, d-raffinose, dl-lactate, erythritol, glycerol, l-arabinose, l-sorbose, lactose, *N*-acetyl-glucosamine and xylitol; and variable for glucuronate. Nitrogen assimilation is negative for nitrate.

The holotype is BCRC 23563^T^, isolated on 23 March 2022 from the cocoon wall of a *P. astacoides blanchardi* (*Coleoptera; Lucanidae*) reared at the Department of Entomology, National Taiwan University, Taipei, Taiwan. It is preserved in a metabolically inactive state as a lyophilized preparation in the BCRC in Hsinchu, Taiwan. The ex-type culture is preserved at the NBRC in Tokyo, Japan, as NBRC 116731, and at the NCYC in Norwich, UK, as NCYC 4470. The GenBank/EMBL/DDBJ accession numbers for the sequences of the D1/D2 domains of the LSU rRNA gene and ITS regions determined in this study are PQ349791 and PQ349796, respectively. The MycoBank accession number is 857608.

## Abbreviations

BCRC: Bioresource Collection and Research Center
NBRC: NITE Biological Resource Center
NCYC: National Collection of Yeast Cultures
YM: yeast malt
